# Availability of specific tools to assess patient reported outcomes in hip arthroplasty in Spain. Identifying the best candidates to incorporate in an arthroplasty register. A systematic review and standardized assessment

**DOI:** 10.1371/journal.pone.0214746

**Published:** 2019-04-01

**Authors:** Jorge Arias-de la Torre, Elisa Puigdomenech, Jose M. Valderas, Jonathan P. Evans, Vicente Martín, Antonio J. Molina, Nuria Rodríguez, Mireia Espallargues

**Affiliations:** 1 Agency for Heath Quality and Assessment of Catalonia (AQuAS), Barcelona, Spain; 2 CIBER Epidemiology and Public Health (CIBERESP), Madrid, Spain; 3 Institute of Biomedicine (IBIOMED), University of León, León, Spain; 4 eHealth Lab Research Group, School of Health Sciences, Universitat Oberta de Catalunya, Catalonia, Spain; 5 Health Services and Policy Research Group, University of Exeter Medical School, Exeter, United Kingdom; 6 Royal Devon and Exeter NHS Foundation Trust, Exeter, United Kingdom; 7 Health Services Research on Chronic Patients Network (REDISSEC), Madrid, Spain; Consorci Parc de Salut MAR de Barcelona, SPAIN

## Abstract

**Purpose:**

1) To systematically review the available scientific literature regarding specific instruments developed and/or tested in a Spanish population, to assess these PROMs in hip arthroplasty; 2) to carry out a standardized assessment of their measurement properties; and 3) to identify the best tools for use in Spain in an arthroplasty registry context.

**Methods:**

A systematic review of PubMed/MEDLINE and EMBASE and CINHAL was done. Furthermore, a standardized assessment of the questionnaires identified using the Evaluating the Measurement of Patient-Reported Outcomes (EMPRO) tool was performed. All developments, validation and studies aiming to assess the measurement properties of PROMs in hip arthroplasty in the Spanish population were included. Data from the questionnaires on metric properties was taken into account to identify the best candidates for inclusion in a register.

**Results:**

A total of 853 documents were found. After screening title and abstract, 13 full text documents were reviewed and 8 questionnaires adapted and validated to assess some of the aspects of hip arthroplasty in the Spanish population were identified. After the EMPRO assessment, 4 questionnaires showed suitable properties (WOMAC, OAKHQOL, mini-OAKHQOL and PFH).

**Conclusions:**

In Spain, there are a few suitable hip-specific questionnaires currently available to assess PROMs in hip arthroplasty surgery. Some of the more widely used questionnaires, like the OHS and HOOS, have not been validated in the Spanish population until now. Identified tools are suitable for use in a clinical context, however their use in an arthroplasty register is more questionable due to the lack of validation studies of the widely used tools in other registers.

## Background

Hip arthroplasties are presently one of the most frequent elective surgeries worldwide. The evidence related to these surgeries points to a significant improvement in the physical function and health-related quality of life of the individuals, as well as the cost-effectiveness and long-term results of these procedures [1,2].

Traditionally, studies on hip arthroplasty surgery have focused on different outcomes, commonly related to patient survival and type of prosthesis used for the arthroplasty [3,4]. These studies usually aimed to assess the mortality rate of patients with a specific type of prosthesis or the revision risk of their prosthesis. Despite this, when assessing the results of a hip arthroplasty, evaluating Patient-Reported Outcome Measures (PROMs) such as quality of life, pain or physical function, among others [3,5–7] has gained importance in recent years, since they can provide different aspects not covered by traditional outcomes like patient-centred care, clinical decision-making or their possible implications in health policy.

There is currently a consensus, both in clinical and in research communities regarding the importance of considering PROMs in hip arthroplasties before and after surgery [2,6,8,9]. In the context of arthroplasty registers, this consensus is made explicit with the continuous incorporation and development of PROMs programs in registers around the world [10,11]. However, this consensus is not such for the most suitable tools to assess these PROMs [12]. As seen from the conclusions of several systematic reviews, until now there have been differences regarding the questionnaires recommended and used, in terms of the quality of different instruments [13–17] and the tools used by arthroplasty registries and clinical trials [10,11].

The instruments used to assess PROMs in hip arthroplasties could be classified in terms of the population they focus on. Specific tools designed to be used solely in populations with hip pathology, e.g. Oxford hip Score (OHS), Hip disability and Osteoarthritis Outcome Score (HOOS), or Harris Hip Score (HHS); and general tools designed for use by the population as a whole, e.g. Short Form-12 or 36 (SF-12 or SF-36), EuroQol-5D (EQ-5D), or World Health Organization Quality of Life Instrument (WHO-QoL) [6,13,14,18–20]. Some of these tools have been deemed suitable measurement properties for use in hip arthroplasty populations worldwide [10,13,14]. In spite of this evidence, and bearing in mind the differences in results among high quality reviews, particularly with specific tools [13–15,17,21], it might be useful to carry out a standardized assessment and systematic comparison when choosing the best tool possible, depending on its context of use. On one hand, a standardized assessment and comparison could be useful to recommend a concrete tool that assesses PROMs in a clinical context, while on the other hand, it might be useful to select the most appropriate tool to be included in an arthroplasty register [22,23].

Focusing on hip-specific PROMs, their selection is generally context-dependent and guided by different criteria of which could be remarkable, among others, the tradition of use in a specific context or country or the availability of a tool [11]. Despite the possible high quality of the selected tools, these criteria are not generally based on a systematic review and comparison of the properties of the tools. Thus, to perform systematic reviews and standardised comparisons of these tools for specific contexts, it could be highly valuable to the decision making process when selecting a questionnaire to assess PROMs in hip arthroplasty.

Therefore, the aims of this study are: 1) to systematically review the scientific literature available about specific instruments developed and/or tested in a Spanish population and Spanish language, to assess PROMs in hip arthroplasty; 2) to evaluate and carry out a standardized assessment of their measurement properties; and 3) to identify the best candidates to be used in an arthroplasty registry context in Spain.

## Methods

To find relevant studies on available hip-specific tools in the Spanish population, a systematic review was performed in accordance with Preferred Reporting Items for Systematic Reviews and Meta-Analyses (S1 Checklist) guidelines [24] and registered with PROSPERO under registry number CRD42018083626 [25].

The search was done up to 01/01/2018 and there was no restriction on the start date. Searches were conducted in the following databases: PubMed/MEDLINE, EMBASE and CINAHL. They were done using a filter developed specifically for PubMed/MEDLINE, which was then adapted for the other databases (S1 Filter). In addition, a manual search was conducted to retrieve any studies that might not have been included in the review. The search strategy and filter development was guided by previous systematic review filters used to select PROMs tools and the Spanish population [13,14,26,27]. Furthermore, references of the retrieved articles were screened for relevant studies, and relevant authors identified in the developmental studies of the different tools were contacted. After the systematic review, the tools assessed in the studies were identified and a standardized assessment of the adequacy of their measurement properties was done using the Evaluating the Measurement of Patient-Reported Outcomes (EMPRO) tool.

### Study inclusion and exclusion criteria and the review process

Population, Intervention, Comparison, Outcome (PICO) criteria were used. All development, adaptation, validation and studies aimed to assess the metric properties of procedure- or condition-specific tools used to assess PROMs in hip arthroplasty in the Spanish population were included. The following exclusion criteria were used: studies published in a language other than English or Spanish, studies focused on general tools, studies not focused on patients undergoing a hip arthroplasty or on the waiting list for one (e.g. studies focused only on osteoarthritis of the hip and not on hip arthroplasty), studies where it was impossible to determine the joint operated on, surgical technique papers and case studies or studies involving fewer than 10 patients. Due to the cross-cultural adaptation and the possible bias related to the comparability of metric properties between populations, only full-texts of tools developed and/or tested in the Spanish population (from Spain) were included [28–30].

After identifying the studies, a screening was done based on the outlined inclusion/exclusion criteria, first by title and abstract and then by full text. All papers that did not meet the inclusion criteria were excluded from the review. All documents identified were revised independently by 2 expert reviewers (JA and JE). If the reviewers did not reach an agreement regarding one or more papers or attributes, a third reviewer (JMV) revised the documents and assessed the questionnaires to arrive at a consensus. After the revision, a standardized assessment of metric properties of the identified tools was done by 2 reviewers (JA and EP) using the EMPRO tool. Before the review, the comprehension of the research aims was assessed using a 10-study pilot and measured using Cohen’s Kappa statistic. None of the reviewers was involved in developing the measures assessed.

### Data extraction and standardized evaluation of proms questionnaires

Data about the instruments identified in the studies included after the screening was retrieved. Information regarding their measurement properties was considered, following the Medical Outcomes Trust classification [31]. In addition, a narrative synthesis of the evidence found in each questionnaire was performed. The quality of the identified questionnaires was assessed and compared based on the evidence found in the studies included.

For the standardized assessment of the questionnaires, the EMPRO tool was used [22]. EMPRO is a tool designed for the standardized evaluation of the quality of instruments used to assess PROMs, based on the Medical Outcomes Trust criteria [31,32]. The questionnaire consists of 39 Likert-type items with a response scale from 1 (“strongly disagree”) to 4 (“strongly agree”) that are distributed among 8 attributes described in Table 1: Conceptual and measurement model (7 items); Reliability (8 items); Validity (6 items); Responsiveness (3 items); Interpretability (3 items); Administration burden (7 items); Alternative modes of administration (2 items); and Cross-cultural and linguistic adaptations (3 items). Additionally, the questionnaire provides a space for comments and references in each item and some items have the response option ‘‘no information” or ‘‘not applicable” when the information is insufficient or not suitable. To conclude the questionnaire, an overall recommendation is provided on a scale with the following response categories: “Strongly recommended”, “Recommended with provisos or alterations”, “Would not recommend” and “Unsure” with a rationale for the recommendation. A score for each attribute was calculated using the mean of the responses to all items composing that attribute and a linear transformation was done to obtain a score from 0 (the worst possible score) to 100 (the best possible score). Finally, an overall score for the questionnaire based on the mean score of the metric-related attributes (conceptual and measurement model, reliability, validity, responsiveness and interpretability) was obtained.

**Table 1:** Attributes assessed using the Evaluating the Measurement of Patient-Reported Outcomes (EMPRO) tool.

Attribute	Definition	Items included
**Conceptual and measurement model**	The rationale for and description of the concept and the populations that a measure is intended to assess and the relationship between these concepts.	1. Concept of measurement stated 2. Obtaining and combining items described 3. Rationality for dimensionality and scales 4. Involvement of target population 5. Scale variability described and adequate 6. Level of measurement described 7. Procedures for deriving scores
**Cultural adaptation **	Cultural and linguistic adaptation of the instrument.	8. Linguistic equivalence 9. Conceptual equivalence 10 Differences between the original and the adapted versions
**Reliability**	The degree to which an instrument is free from random error.	Internal consistency: 11. Data collection methods described 12. Cronbach’s alpha adequate 13. IRT estimates provided 14. Testing in different populations Reproducibility: 15. Data collection methods described 16. Test–retest and time interval adequate 17. Reproducibility coefficients adequate 18. IRT estimates provided
**Validity**	The degree to which the instrument measures what it purports to measure.	19. Content validity adequate 20. Construct/criterion validity adequate 21. Sample composition described 22. Prior hypothesis stated 23. Rational for criterion validity 24. Tested in different populations
**Responsiveness**	An instrument’s ability to detect change over time.	25. Adequacy of methods 26. Description of estimated magnitude of change 27. Comparison of stable and unstable groups
**Interpretability**	The degree to which one can assign easily understood meaning to an instrument’s quantitative scores.	28. Rational of external criteria 29. Description of interpretation strategies 30. How data should be reported stated
**Burden**	The time, effort, and other demands placed on those to whom the instrument is administered (respondent burden) or on those who administer the instrument (administrative burden).	Respondent: 31. Skills and time needed 32. Impact on respondents 33. Not suitable circumstances Administrative: 34. Resources required 35. Time required 36. Training and expertise needed 37. Burden of score calculation
**Alternative modes of administration**	Alternative modes of administration used for the administration of the instrument	38. The metric characteristics and use of each alternative mode of administration 39. Comparability of alternative modes of administration

## Results

**Fig 1:**
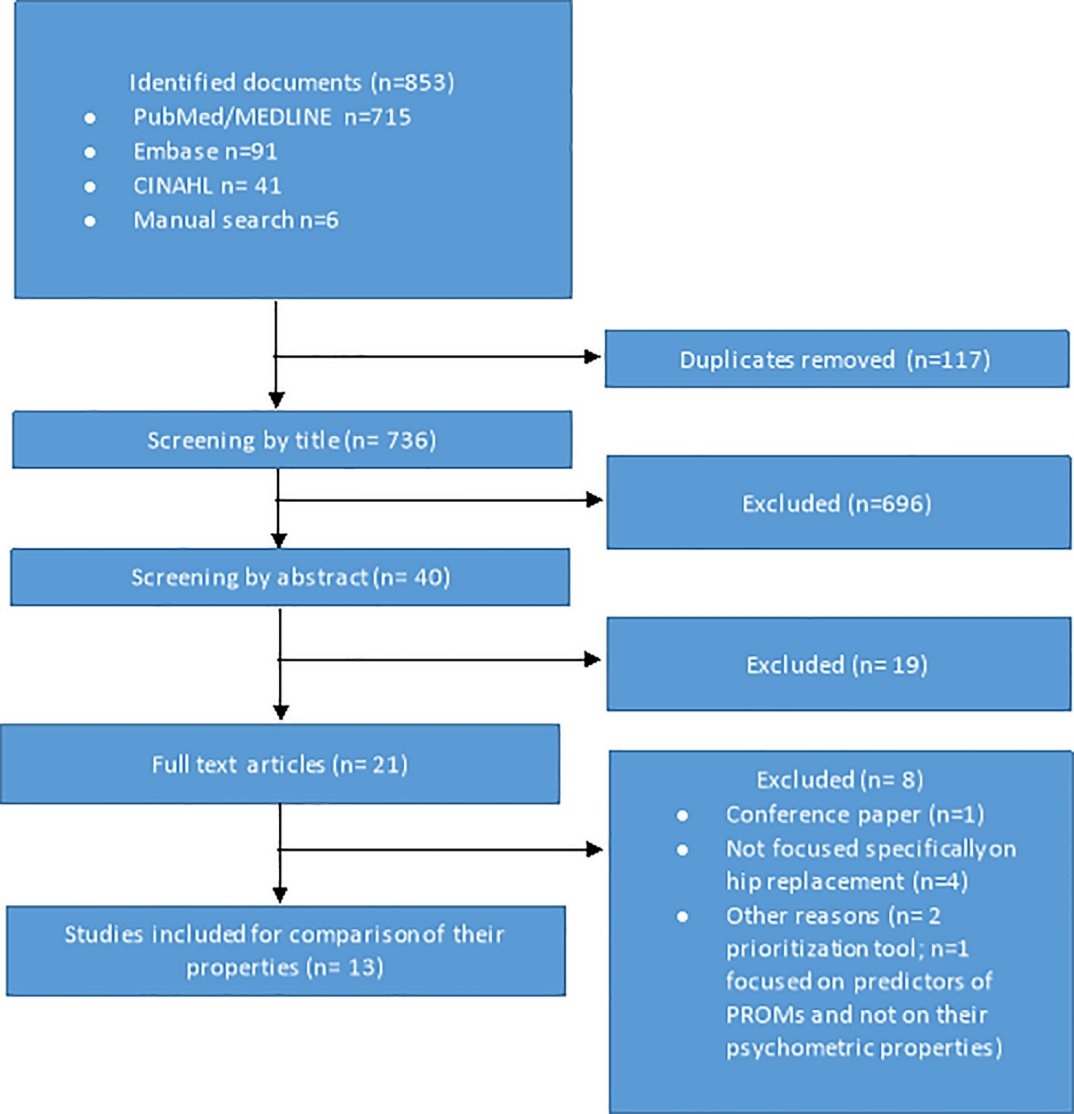
Review process. Flow diagram.

Fig 1 shows a flow diagram of the review process. Using the filter developed for systematic review, a total of 853 documents were identified. Of these, 715 (83.8%) were identified in PubMed/MEDLINE, 91 (10.7%) in EMBASE, 41 (4.8%) in CINAHL and 6 (0.7%) through a manual search. After checking for duplicates, 117 (13.7%) were removed. Of the remaining 736 titles, 696 were excluded, leaving a total of 40 abstracts to screen. After screening the abstracts, 21 full text articles were considered. Of these documents, 8 were excluded: 1 was a conference abstract, 4 were focused on hip osteoarthritis without considering hip replacement or it was impossible to determine the operated joint, 2 aimed to establish prioritisation systems rather than assess PROMs in hip replacement, and 1 was focused on variables that might act as predictors of PROMs and not on their specific measurement properties. Finally, a total of 13 full text articles published between 1997 and 2017 were included in the data extraction [33–45].

Taking into account the instruments included in the studies (Table 2), a total of 8 tools were identified, four of which were variants or sub-scales of other main tools. In terms of specific questionnaires, we found: The Western Ontario and McMaster Universities Osteoarthritis Index (WOMAC) [33–36,44], composed by 24 Likert-type items designed to assess pain, stiffness and physical function; two different versions of the WOMAC Short Form, each composed of 11 items with 5 Likert-type items [35,37]; The WOMAC Short Form (Function dimension) composed of 7 items validated to assess functionality in joint replacement [38]; The Pain and Function of the Hip scale (PFH) composed of 9 Likert-type items to assess pain, function and mobility/strength [41,42,45]; The Osteoarthritis Knee and Hip Quality of Life (OAKHQOL) composed of 43 Likert-type items, 40 of which are used to assess physical activity, mental health, pain, social support and social functioning [39]; The Mini-OAKHQOL composed of 20 Likert-type items to assess the same dimensions as the long version (OAKHQOL); and the Harris Hip Score (HHS), with 10 Likert-type items that assess pain, function, amplitude of movement and absence of deformity [43].

**Table 2:** General characteristics of the instruments identified from validation studies in hip arthroplasty population.

Questionnaire	Author (year)	Number of items and type	Assessed dimensions (number of items)	Punctuation
Western Ontario and McMaster Universities Osteoarthritis Index (WOMAC)	Escobar et al. (2002) Quintana et al. (2005) Lopez Alonso et al. (2009) Escobar et al. (2012) Quintana et al (2012)	24 Likert-type items with a 5-point response scale	Pain (5 items), stiffness (2 items) and physical function (17 items)	Standardized from 0 (best health status) to 100 (worst health status)
WOMAC Short Form (v1)	Bilbao et al. (2011)	11 Likert-type items with a 5-point response scale	Pain (3 items) and physical function (8 items)	Standardized from 0 (best health status) to 100 (worst health status)
WOMAC Short Form (v2)	Lopez Alonso et al. (2009)	11 Likert-type items with a 5-point response scale	Symptomatology and physical disability in: repose (4 items), movement on stairs (3 items), put on or take off shocks (2 items), and Stiffness (2)	NS
WOMAC (Short Form. Function dimension)	Escobar et al. (2011)	7 Likert-type items with a 5-point response scale	Functional capacity (capacity to do activities)	Standardized from 0 (best health status) to 100 (worst health status)
Osteoarthritis Knee and Hip Quality of Life (OAKHQOL)	Gonzalez Saenz de Tejada et al. (2011)	43 Likert-type items with a 10-point response scale	Physical activity (16 items), mental health (13 items), pain (4 items), social support (4 items), and social functioning (3 items) and three independent items addressing sex life, professional life and fear of being dependent	Standardized from 0 (worst quality of life) to 100 (best quality of life)
Mini-OAKHQOL	Gonzalez Saenz de Tejada et al. (2017)	20 Likert-type items with a 10-point response scale	Physical activity (7 items), mental health (3 items), pain (3 items), social support (2 items), and social functioning (2 items) and three independent items addressing sex life, professional life and fear of being dependent	Standardized from 0 (worst quality of life) to 100 (best quality of life)
Pain and Function of the Hip scale (PFH)	Valls et al. (1997) Alonso et al. (2000) Marti-Valls et al. (2000)	9 Likert-type items with different point response scales depending on the specific dimension.	Pain (2 items), function (3 items) and mobility/Strength (4 items)	From 0 (total functional limitation) to 85 (absence of functional limitation)
Harris Hip Score (HHS)	Navarro Collado et al. (2005)	10 Likert-type items (different points for each response scale)	Pain (1 item), function (7 items), amplitude of movement (1 item) and absence of deformity (2 items)	From 0 (worst possible functional capacity) to 100 (best possible functional capacity)

v1: version 1; v2: version 2; NS: Not explicitly specified

Considering the properties of the identified tools, Table 3 shows their adaptation according to the EMPRO standardized assessment. Based on the overall score, it was observed that only OAKHQOL, mini-OAKHQOL, WOMAC and PFH could be considered as reasonably acceptable (EMPRO overall score > = 50). The highest overall score corresponded to WOMAC with a score of 65.47, followed by OAKHQOL with a score of 58.87, mini-OAKHQOL with 58.57 points and PFH with 50.71 points. In addition, focusing on the specific metric attributes it was observed that all the assessed tools have EMPRO scores equal to or higher than 50 in reliability, validity and responsiveness, except PFH in validity. Only WOMAC and PFH had a score equal to or higher than 50 points in conceptual and measurement model and in interpretability. Regarding non-metric attributes (cultural adaptation and burden), only OAKHQOL had a score equal to or higher than 50 points.

**Table 3:** Attributes of each of the questionnaires identified from the studies included in the systematic review. EMPRO.

Questionnaire	Conceptual and measurement model	Cultural adaptation	Reliability	Validity	Responsiveness	Interpretability	Burden	Alternative modes of administration	Overall score
WOMAC	++	+	++	++	+++	++	+	+	65.47
WOMAC SF (v1)	+	+	++	++	++	+	+	-	48.99
WOMAC SF (v2)	+	+	++	+	+	+	+	-	-
WOMAC SF (FD)	+	+	++	+	+++	+	+	-	-
OAKHQOL	+	+++	++	++	+++	+	++	-	58.87
Mini-OAKHQOL	+	+	+++	++	+++	+	+	-	58.57
PFH	++	+	++	+	++	++	+	-	50.71
HHS	+	+	+	+	++	+	+	-	-

WOMAC: Western Ontario and McMaster Universities Osteoarthritis Index; SF: Short form; v1: version 1; v2: version 2; FD: Function dimension; OAKHQOL: Osteoarthritis Knee and Hip Quality of Life; PFH: Pain and Function of the Hip Scale; HHS: Harris Hip Score

+: EMPRO score 25–49; ++: EMPRO score 50–74; +++: EMPRO score 75–100; -: EMPRO score not applicable or not calculable

## Discussion

Nowadays in Spain, the availability of specific tools to assess PROMs in hip arthroplasty is limited. After the systematic review of the available literature, only eight instruments (including short forms) were identified. Of these instruments only WOMAC, OAKHQOL, mini-OAKHQOL, and PFH, have shown suitable measurement properties to be used in the Spanish population. In addition, none of the most widely used hip-specific questionnaires was identified in arthroplasty registers around the world [10,11,21]. This evidence suggests that there are some suitable tools to be included in an arthroplasty register currently in Spain but, before deciding to do so, it might be valuable to carry out validation studies of widely used hip-specific tools in other registers worldwide as OHS and HOOS.

Taking into account the specific tools assessed in our study, only 4 were identified as suitable candidates and 1 of them, mini-OAKHQOL, was a shorter version of one of the main tools. Of these tools, the most acceptable, according to EMPRO guidelines, was WOMAC, which was the only tool used by 4 other arthroplasty registers worldwide [11]. In addition, the similarity between the EMPRO overall scores from OAKHQOL and the shorter version should be highlighted. Given the similarity in metric properties, we could argue that it would be better to use the mini-OAKHQOL tool over the full version since it has a lower burden, but this hypothesis requires more evidence to support it.

Several reviews assessing the acceptability of instruments to evaluate PROMs in hip arthroplasty were previously done in other countries [13–15,17]. From these reviews, specific instruments used with better properties were identified, which in most cases were the OHS and the HOOS. In addition, the most common instruments used in other registries were also the OHS and HOOS [10,21].

Regarding OHS, this questionnaire is one of the most widely used for different reasons, among which could be highlighted its length, including only 12 items, and its acceptable metric properties to be used in a wide range of languages and countries. Despite this, for the Spanish population, only one validation study was found [46]. This study was condition-focused and not procedure-focused, which was why we decided to exclude it. Patient characteristics of the population included in this study might be different from arthroplasty patients. For this reason, and bearing in mind one of the objectives was to select the best candidate tool to be included in an arthroplasty register, we decided to discard this study, again showing the need for procedure-focused validation studies of this questionnaire. The HOOS questionnaire is also one of the most widely used questionnaires in hip arthroplasties, used by the Swedish Arthroplasty Register among others [47], and has shown its acceptability in metric terms in other contexts [13,14,18,20,48]. With these reasons in mind, we propose that the adaptation and validation of OHS and HOOS in the hip arthroplasty population could be valuable in our context, both for clinical use and to know what could be the most acceptable tool for inclusion in an arthroplasty register.

Finally, and despite the suitability of the metric properties of all tools in which was possible to calculate an EMPRO overall score, except WOMAC Short Form whose score was on the border of acceptability, we should highlight that they might only be considered as partially suitable candidates for inclusion in an arthroplasty register. As these tools are not among those most used by other registries, it is difficult to pool data and compare them. Additionally, when the properties of PROMs tools were compared in other contexts and countries [13,14,17], these tools were not present in some cases, OAKHQOL mini-OAKQOL and PFH, for example, or they were not the best candidates for adequacy or specificity. On the other hand, focusing on WOMAC, it should also be noted that the full questionnaire is included in HOOS, which is one of the more widely used questionnaires to assess PROMS in hip arthroplasties [14,20,48,49].

Some limitations of this review should be discussed. Firstly, we should mention that we only examined tools developed and/or tested in the population from Spain. This inclusion criteria, seems to limit the scope of the study. In spite of this, we think that with this approach, our results are much more accurate. Thus, we deem that they could be much more useful for the clinical community of Spain and, furthermore, they may serve as baseline for future comparative studies between countries. Besides, we have to remark the possible publication bias that could affect the results shown. Studies about PROMs could be unpublished or published as grey literature. Despite this, we have tried to be as exhaustive as possible in the literature search and have also tried to contact the most influential authors. As such, we feel that we have identified at least the main validation literature focused on hip arthroplasty specific tools. Another limitation is that we have excluded studies focused on patients with OA if the patients included in the studies were not on a waiting list or undergoing hip arthroplasty. Though OA is by far the most common cause of total hip replacement, it should be noted that not all patients affected by OA are eligible candidates for an arthroplasty [50–53]. OA is a chronic condition that could affect people with very different characteristics, including young patients and patients with non-severe OA. Young patients able to perform their daily activities well should not be eligible for an arthroplasty, and especially not for a total hip replacement. For these reasons, we consider the outlined inclusion and exclusion criteria appropriate to address the aims of this study. In addition, we want to point out the limitation related to including only hip specific tools and not general tools. General instruments are widely studied and should be used as the first approach to PROMs assessment. Nevertheless, and despite their use for assessment of specific populations, these instruments were not specifically designed to assess PROMs, which means their responsiveness could be compromised. Furthermore, in some cases the constructs evaluated with general tools are not the same or not entirely equivalent than those evaluated by the specific tools. After taking the conceptual differences between general and specific tools into account, particularly when used in specific populations, we decided to exclude general tools. Finally, we should highlight the lack of validation studies developed from the Item Response Theory (IRT) perspective. Including this approach when evaluating the metric properties of questionnaires could improve the precision of their assessment and decision making when selecting a specific tool.

In conclusion, our study shows that currently in Spain, there are some specific questionnaires with adequate metric properties to assess PROMs in hip arthroplasty. These tools are: WOMAC, OAKHQOL, mini-OAKHQOL and PFH. While these tools might be considered suitable for use in a clinical context, their recommendation for use in an arthroplasty register is more questionable, mainly due to the lack of validation studies of the OHS and HOOS, the more widely used tools in this context.

## Supporting information

S1 Checklist PRISMA 2009 checklist. (DOC)

S1 Filter PubMed filter. PubMed/MEDLINE filter. Psychometric properties of specific PROMs questionnaires in the Spanish population.
(DOCX)
