# Supplementary material for: Availability of specific tools to assess patient reported outcomes in hip arthroplasty in Spain. Identifying the best candidates to incorporate in an arthroplasty register. A systematic review and standardized assessment
Source: PLoS One. 2019 Apr 1;14(4):e0214746. doi: 10.1371/journal.pone.0214746 (PMC6443164; doi:10.1371/journal.pone.0214746)
Supplement: S1 Filter — PubMed/MEDLINE filter. Psychometric properties of specific PROMs questionnaires in the Spanish population. (DOCX) [file pone.0214746.s002.docx]

**S1 filter. PubMed/MEDLINE filter. Psychometric properties of specific PROMs questionnaires in the Spanish population**

**#1: Construct search**

(HR-PRO OR HRPRO OR HRQL OR HRQoL OR QL OR QoL OR quality of life OR (health index* OR health indices or health profile*) OR health status OR ((patient or self OR carer OR proxy) adj (appraisal* or appraised OR report OR reported OR reporting OR rated OR rating* OR based OR assessed OR assessment*)) OR (disability or function OR functional OR functions OR subjective OR utility OR utilities OR wellbeing or well being OR priorit* OR waiting))

**#2: population search**

(hip[tiab] OR hip[MeSH]

**#3: instrument search**

(index OR indices OR instrument OR instruments OR measure OR measures OR questionnaire* OR profile OR profiles OR scale OR scales OR score OR scores OR status OR survey OR surveys)

**#4 geographical search**

(Spain or Spanish or (Spanish adj version) or (Spanish adj validation) or (Spanish adj translation) or (Crosscultural adj adaptation) or (Cross-cultural adj validation) or (catalunya OR catalonia OR cataluna OR catala* OR barcelon* OR tarragona OR lleida OR lerida OR girona OR gerona) OR (valencia* OR castello* OR alacant OR alicant*) OR (murcia*) OR (andalu* OR sevill* OR granad* OR huelva OR almeria OR cadiz OR jaen OR malaga OR (cordoba NOT argentin*) OR (extremadura OR caceres OR Badajoz) OR (madrid OR madri*) OR (castilla OR salamanca OR zamora OR valladolid OR segovia OR soria OR palencia OR avila OR burgos) OR (leon NOT (france OR clermont OR rennes OR lyon OR USA OR mexic*)) OR (galicia OR gallego* OR compostela OR vigo OR corun* OR orense OR ourense OR pontevedra OR lugo) OR (oviedo OR gijon OR asturia*) OR (cantabr* OR santander) OR (vasco OR euskadi OR basque OR bilbao OR donosti* OR san sebastian OR vizcaya OR bizkaia OR guipuzcoa OR gipuzkoa OR alava OR araba OR vitoria OR gasteiz) OR (navarra OR nafarroa OR pamplona OR iruna OR irunea) OR (logron* OR rioj*) OR (aragon* OR zaragoza OR teruel OR huesca) OR (mancha OR ciudad real OR albacete OR cuenca) OR (toledo NOT (ohio OR us OR usa OR OH)) OR (guadalajara NOT mexic*) OR (balear* OR mallorca OR menorca OR ibiza OR eivissa) OR (canari* or palmas OR lanzarote OR canary* OR tenerif*) OR (ceuta OR melilla)))

**#4: 1 AND #2 AND #3 AND filter for measurement properties**

Validation Studies[pt] OR Comparative Study[pt] OR “psychometrics”[MeSH] OR psychometr*[tiab] OR clinimetr*[tw] OR clinometr*[tw] OR “outcome assessment (health care)”[MeSH] OR outcome assessment[tiab] OR outcome measure*[tw] OR “observer variation”[MeSH] OR observer variation[tiab] OR “Health Status Indicators”[Mesh] OR “reproducibility of results”[MeSH] OR reproducib*[tiab] OR “discriminant analysis”[MeSH] OR reliab*[tiab] OR unreliab*[tiab] OR valid*[tiab] OR coefficient[tiab] OR homogeneity[tiab] OR homogeneous[tiab] OR “internal consistency”[tiab] OR (cronbach*[tiab] AND (alpha[tiab] OR alphas[tiab])) OR (item[tiab] AND (correlation*[tiab] OR selection*[tiab] OR reduction*[tiab])) OR agreement[tiab] OR precision[tiab] OR imprecision[tiab] OR “precise values”[tiab] OR test–retest[tiab] OR (test[tiab] AND retest[tiab]) OR (reliab*[tiab] AND (test[tiab] OR retest[tiab])) OR stability[tiab] OR interrater[tiab] OR inter-rater[tiab] OR intrarater[tiab] OR intra-rater[tiab] OR intertester[tiab] OR inter-tester[tiab] OR intratester[tiab] OR intra-tester[tiab] OR interobserver[tiab] OR inter-observer[tiab] OR intraobserver[tiab] OR intra-observer[tiab] OR intertechnician[tiab] OR inter-technician[tiab] OR intratechnician[tiab] OR intra-technician[tiab] OR interexaminer[tiab] OR inter-examiner[tiab] OR intraexaminer[tiab] OR intra-examiner[tiab] OR interassay[tiab] OR inter-assay[tiab] OR intraassay[tiab] OR intra-assay[tiab] OR interindividual[tiab] OR inter-individual[tiab] OR intraindividual[tiab] OR intra-individual[tiab] OR interparticipant[tiab] OR inter-participant[tiab] OR intraparticipant[tiab] OR intra-participant[tiab] OR kappa[tiab] OR kappa’s[tiab] OR kappas[tiab] OR repeatab*[tiab] OR ((replicab*[tiab] OR repeated[tiab]) AND (measure[tiab] OR measures[tiab] OR findings[tiab] OR result[tiab] OR results[tiab] OR test[tiab] OR tests[tiab])) OR generaliza*[tiab] OR generalisa*[tiab] OR concordance[tiab] OR (intraclass[tiab] AND correlation*[tiab]) OR discriminative[tiab] OR “known group”[tiab] OR factor analysis[tiab] OR factor analyses[tiab] OR dimension*[tiab] OR subscale*[tiab] OR (multitrait[tiab] AND scaling[tiab] AND (analysis[tiab] OR analyses[tiab])) OR item discriminant[tiab] OR interscale correlation*[tiab] OR error[tiab] OR errors[tiab] OR “individual variability”[tiab] OR (variability[tiab] AND (analysis[tiab] OR values[tiab])) OR (uncertainty[tiab] AND (measurement[tiab] OR measuring[tiab])) OR “standard error of measurement”[tiab] OR sensitiv*[tiab] OR responsive*[tiab] OR ((minimal[tiab] OR minimally[tiab] OR clinical[tiab] OR clinically[tiab]) AND (important[tiab] OR significant[tiab] OR detectable[tiab]) AND (change[tiab] OR difference[tiab])) OR (small*[tiab] AND (real[tiab] OR detectable[tiab]) AND (change[tiab] OR difference[tiab])) OR meaningful change[tiab] OR “ceiling effect”[tiab] OR “floor effect”[tiab] OR “Item response model”[tiab] OR IRT[tiab] OR Rasch[tiab] OR “Differential item functioning”[tiab] OR DIF[tiab] OR “computer adaptive testing”[tiab] OR “item bank”[tiab] OR “cross-cultural equivalence”[tiab])

**#5: #4 AND (English[Language] or Spanish[Language])**

**#6: #5 NOT** (“animal” [MeSH Terms])
